# Deep Learning for Localization Microscopy in 2D and 3D

**DOI:** 10.1021/acs.accounts.6c00345

**Published:** 2026-07-24

**Authors:** Ofri Goldenberg, Dafei Xiao, Yoav Shechtman

**Affiliations:** † Faculty of Biomedical Engineering, Technion−Israel Institute of Technology, Haifa 3200003, Israel; ‡ Russell Berrie Nanotechnology Institute, Technion−Israel Institute of Technology, Haifa 3200003, Israel; § Faculty of Electrical and Computer Engineering, Technion−Israel Institute of Technology, Haifa 3200003, Israel

## Abstract

Super-resolution microscopy has fundamentally transformed our ability to observe biological structures, allowing nanoscale observation into biological samples such as fixed and live cells and tissues, and has been notably recognized by the 2014 Nobel Prize in Chemistry. Single-molecule localization microscopy (SMLM) stands out as a highly successful and widely accessible method for super-resolution microscopy. However, because SMLM relies on computationally reconstructing a single image from thousands of sparse frames, it suffers from significant algorithmic bottlenecks, particularly when dealing with high emitter densities or three-dimensional data. Deep learning has emerged as an exceptionally effective solution to bypass these computational challenges, enabling fast, parameter-free reconstruction. The application of neural networks for SMLM analysis is uniquely powerful because such networks can be trained entirely on simulated data; since the optical point spread function is well-understood, and SMLM images are fundamentally simple, consisting of a collection of point-spread functions, it is relatively easy to numerically simulate the vast quantities of highly accurate training images required to train reconstruction nets.

In this Account, we summarize our contributions to localization microscopy by applying neural nets to address its limitations and bottlenecks. Specific challenges and applications include dense molecule fitting in 2D and in 3D, single-channel multicolor imaging, super spatiotemporal resolution microscopy, optical genome mapping, large field-of-view (FOV) imaging, and accurate background simulation. The fact that the training of the neural net is based on simulated images enables powerful and interesting capabilities. For example, the optical setup itself can be algorithmically designed together with the decoding neural net; we have used this concept for tasks such as designing optimal phase masks for depth encoding, for spectral encoding, and even for both simultaneously.

## Graphical abstract

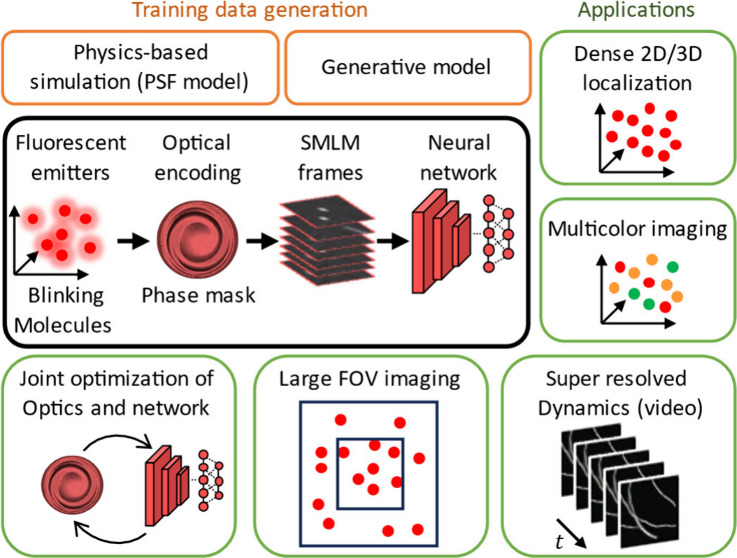

## Key References

-

Nehme, E. ; Weiss, L. E. ; Michaeli, T. ; Shechtman, Y. Deep-STORM: Super-Resolution Single-Molecule Microscopy by Deep Learning. Optica 2018, 5, 4, 458–464 .^1^ A neural net trained on simulated images can successfully resolve closely spaced emitters with which classical algorithms struggle.
-

Hershko, E. ; Weiss, L. E. ; Michaeli, T. ; Shechtman, Y. Multicolor Localization Microscopy and Point-Spread-Function Engineering by Deep Learning. Opt. Express, OE 2019, 27, 5, 6158–6183 10.1364/OE.27.00615830876208.^2^ Single-channel multicolor imaging of emitters is achieved by optimizing a phase mask that encodes the color of an emitter in the shape of its point-spread function, while jointly training a neural net to decode the information.
-

Nehme, E. ; Freedman, D. ; Gordon, R. ; Ferdman, B. ; Weiss, L. E. ; Alalouf, O. ; Naor, T. ; Orange, R. ; Michaeli, T. ; Shechtman, Y. DeepSTORM3D: Dense 3D Localization Microscopy and PSF Design by Deep Learning. Nat. Methods 2020, 17, 7, 734–740 32541853 10.1038/s41592-020-0853-5PMC7610486.^3^ A neural net can resolve highly dense emitters in 3D, imaged using an optical depth-encoding phase mask, beyond the capabilities of any classical algorithm. Furthermore, the encoding phase mask itself is then algorithmically jointly optimized together with the decoding net.
-

Saguy, A. ; Alalouf, O. ; Opatovski, N. ; Jang, S. ; Heilemann, M. ; Shechtman, Y. DBlink: Dynamic Localization Microscopy in Super Spatiotemporal Resolution via Deep Learning. Nat. Methods 2023, 20, 12, 1939–1948 37500760 10.1038/s41592-023-01966-0.^4^ To improve the temporal resolution of localization microscopy, a neural net is trained to interpolate in space and time and produce super-resolution video from blinking data.

## 1 Introduction

The motivation to observe objects with increasingly smaller details has been a driving force in microscopy throughout history. Already in the 19th century, optical microscopy hit the physical imaging resolution bound dictated by the diffraction limit, of roughly half the wavelength of visible light, which translates in practice to ∼ 200–300 nm. Toward the end of the 20th century/early 21st century, techniques were developed that circumvent this bound; these include structured illumination microscopy (SIM),^5^ stimulated emission depletion (STED)^6^ and SMLM.^7–10^ SMLM is based on recording a movie in which the fluorescent emission from many individual molecules (typically ∼ 10^6^) is captured over many frames (typically ∼ 10^3^–10^5^). The emission is made to be sparse per-frame, by various methods, e.g. relying on stochastic emission from dye molecules^7,11^ or on transient binding of imaging strands that can exhibit either environmentally dependent^10^ or constant^12^ fluorescence. In any of these cases, similar downstream image analysis takes place, consisting of fitting individual molecules and determining their positions, which can be done at very high precision, and then algorithmically combining the information from the entire movie to yield a single super-resolved image at a resolution of tens of nanometers. By beating the diffraction limit by an order of magnitude, allowing researchers to image biological structures at the nanoscale, SMLM had a strong impact on biomicroscopy, and it has been recognized with the 2014 Nobel Prize in Chemistry.

Since the 2010s, machine learning, and specifically deep neural networks, have demonstrated amazing utility for a wide variety of image processing tasks, and microscopy is no exception. Deep neural networks learn directly from data, rather than relying entirely on signal modeling, which is highly advantageous, for example, in cases where modeling is difficult, or where applying a model is computationally resource consuming. Key examples include tasks such as cellular/nucleus segmentation,^13,14^ denoising and deblurring,^15,16^ cell phenotyping,^17^ and more.

Naturally, deep learning became instrumental also in SMLM. Arguably the main enabler for that is the fact that we have very good numerical models for the microscopic Point Spread Function (PSF), and thus it is relatively easy to numerically simulate as many training images as we need, resembling experimental SMLM frames, namely, randomly placed emitters, at densities and signal-to-noise/background levels of our choice. Combined with the powerful capabilities of neural nets when it comes to inverse problems, deep learning is now able to solve problems that were previously unsolvable, e.g. finding the 3D positions and/or emission spectra of multiple emitters that are closely spaced, and more.

In this paper we discuss the principles of 2D and 3D localization microscopy, the integration of deep learning, and the transformative impact of their combination. We briefly review the historical development of SMLM and PSF engineering, outlining the computational bottlenecks and spatiotemporal trade-offs that existed prior to the deep learning era, and discuss how deep neural networks have revolutionized the field, focusing on key achievements by our group.

## 2 Localization Microscopy Before Deep Learning

Localization microscopy, based on determining the positions of individual fluorescent emitters with precision far exceeding the optical diffraction limit, originated in single-particle tracking (SPT), which emerged in the late 1980s and early 1990s as researchers showed that the center position of an individual fluorescent or scattering particle could be estimated with nanometer-scale precision from its diffraction-limited image, provided enough photons were collected.^20–23^ By fitting the image of an isolated particle to a model of the point spread function (typically a two-dimensional Gaussian), early SPT studies could follow the trajectories of individual molecules on cell membranes and motor proteins along cytoskeletal filaments, revealing heterogeneous dynamics invisible to macroscopic population-averaged measurements.^22,24^ Notably, Fluorescence Imaging with One Nanometer Accuracy (FIONA) achieved ∼ 1.5 nm precision to track the discrete steps of molecular motors.^25^ These ideas matured throughout the 1990s and 2000s into a rich methodological landscape for studying diffusion, transport, and molecular interactions in living cells.^26,27^

SMLM transformed the localization principle from a tracking tool into a super-resolution imaging method. The conceptual breakthrough, demonstrated independently by four groups in 2006, was to combine precise single-molecule localization with a mechanism for temporally separating the emission of densely packed fluorophores, by manipulating photophysics. Betzig et al. introduced photoactivated localization microscopy (PALM, Figure 1b), using photoactivatable fluorescent proteins to stochastically switch on sparse subsets of emitters per frame.^8^ Simultaneously, Rust, Bates, and Zhuang demonstrated stochastic optical reconstruction microscopy (STORM, Figure 1a), achieving the same goal using photoswitchable organic dyes,^7^ while Hess, Girirajan, and Mason reported a similar approach termed FPALM.^9^ By accumulating localizations over thousands of frames, these methods reconstructed images with spatial resolution of tens of nanometers, i.e. an order of magnitude beyond the diffraction limit (Figure 1d). The impact of this work, together with the contributions of Stefan Hell to STED microscopy^6^ and W. E. Moerner’s foundational single-molecule detection and imaging studies,^28^ was recognized with the 2014 Nobel Prize in Chemistry. Since then, the SMLM family has expanded to include numerous variants such as DNA-PAINT^12^ (Figure 1c) and related techniques, and has become a central tool in cell biology and biophysics.^29,30^

**Figure 1:**
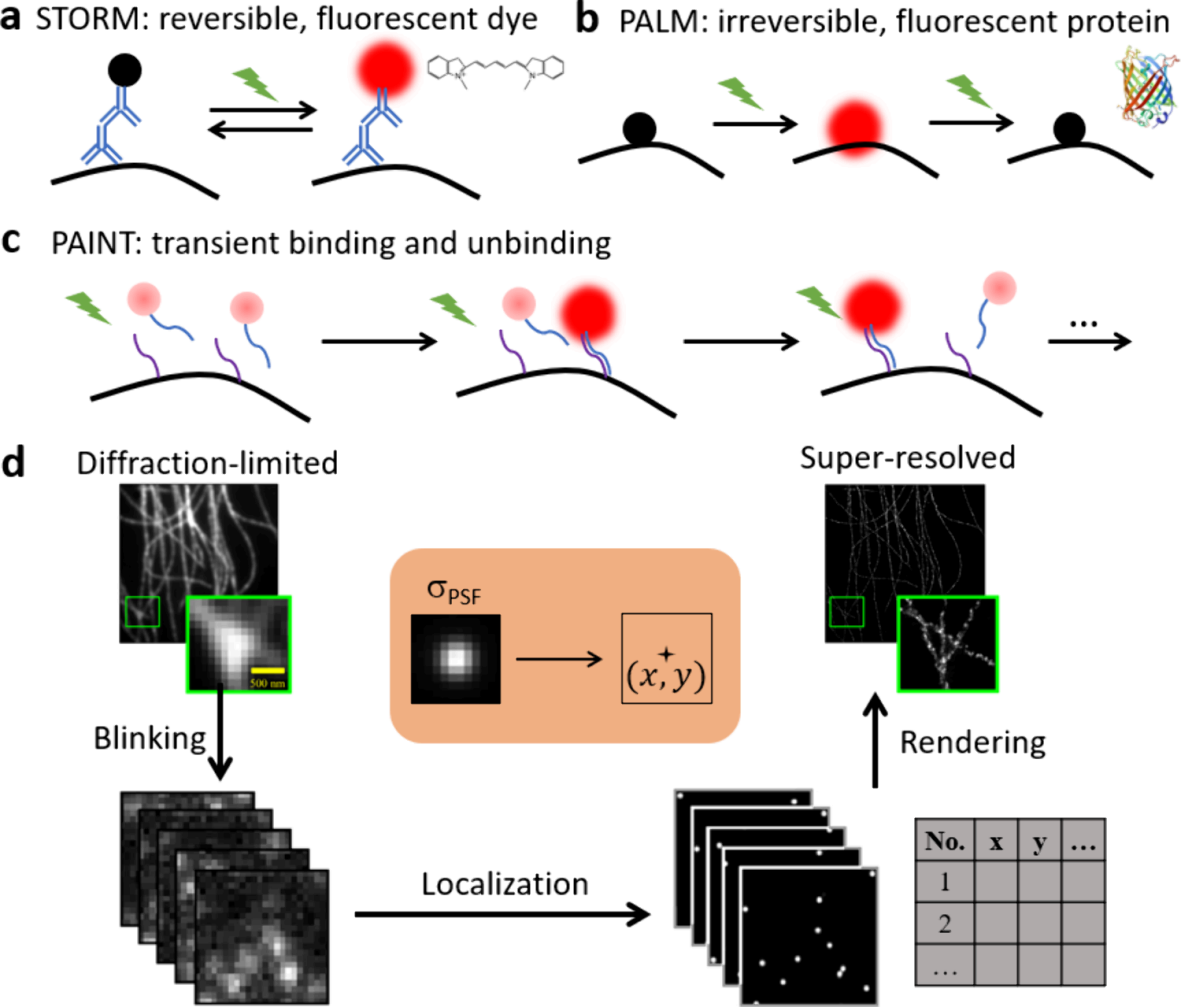
Schematics of representative blinking mechanisms and diffraction-limit breaking in SMLM. (a) STORM blinking based on reversible photoswitching. (b) PALM blinking based on irreversible photoactivation followed by photobleaching. Fluorescent proteins are genetically fused to target proteins and expressed by the cell. GFP Image from the RCSB PDB^18^ of PDB ID 1EMA.^19^ (c) PAINT blinking based on transient binding and unbinding of fluorescent probes to docking sites on the target structure. Here, DNA-PAINT^12^ is depicted, a variant of PAINT where the transient binding is achieved by specific hybridization of short, complementary DNA strands. (d) Principle of super-resolution in SMLM: the diffraction-limited PSF (with a physical extent of *σ*_*PSF*_ which is a few hundred nanometers) is localized with high precision to estimate molecular coordinates. Stochastic blinking separates continuous fluorescence into a sequence of frames with sparse emitters. Frame-by-frame localization yields a set of molecular positions, which are then reconstructed into a super-resolution image. Adapted with permission from ref 1. Copyright 2018 Optical Society of America.

### 2.1 Point-Spread Function Engineering

Extending 2D SMLM to three dimensions can be achieved through PSF engineering. The standard microscope PSF is approximately axially symmetric about the focal plane and rapidly loses SNR with defocus, resulting in ambiguous axial (z) localization and a limited depth range. PSF engineering addresses this limitation by deliberately reshaping the detection PSF, e.g. via a phase mask placed in the pupil plane of a 4f-extension (Figure 2a), to encode axial position into the lateral image of single emitters, enabling 3D localization with a conventional 2D camera.

**Figure 2:**
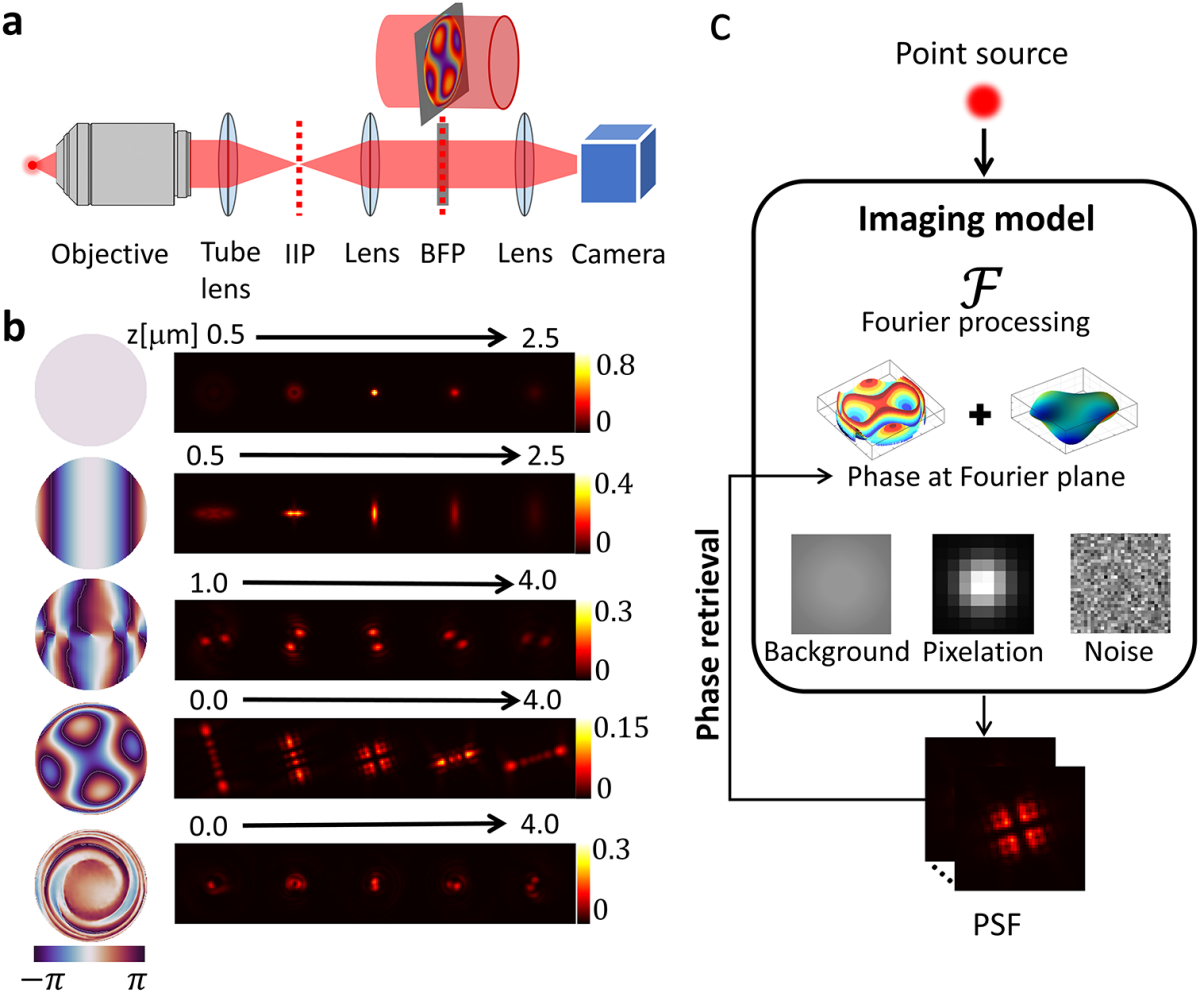
PSF engineering for SMLM. (a) The microscope is extended with a 4f system, in which a phase mask is placed at the back focal plane (BFP). IIP: intermediate image plane. (b) Representative phase masks (from top to bottom, standard, astigmatism, double helix, tetrapod, end-to-end learning) and their corresponding PSF z-stacks. The axial coordinate z denotes the distance between the emitter and the coverslip. Color bars are normalized to the peak intensity of the standard PSF, which is defined as unity. To enhance visual contrast, the color scale for the standard PSFs is truncated at 0.8 rather than extending to the full normalized intensity of 1. (c) Phase-retrieval-based PSF calibration. The imaging process is modeled as Fourier propagation with pupil-phase terms, followed by background, pixelation, and noise. Calibration estimates the pupil phase such that the model PSFs best match the measured data.

Early implementations include the astigmatic PSF (Figure 2b), introduced by inserting a cylindrical lens into the detection path.^31^ This produces an elliptical PSF whose aspect ratio varies monotonically with axial position over ∼ 1 μm with a high-NA objective. Localization is performed by fitting an elliptical 2D Gaussian and mapping the fitted widths to axial position via calibration. While simple and efficient, this approach is limited in axial range and loses accuracy near its boundaries. To extend the depth range, the double-helix (DH) PSF was developed^32^ (Figure 2b), generating two lobes that rotate with axial displacement and encode z-position through their relative angle over several micrometers. Determining the axial position of an emitter originally required identifying both lobes and estimating their relative angle, making the fitting process more complex and susceptible to errors when emitters overlap.

The question of the optimal PSF for single-particle localization led to the development of Tetrapod PSFs^33,34^ (Figure 2b), which were derived to be maximally informative, in the sense of Fisher information. These pupil-phase-engineered PSFs generate complex multilobe patterns that evolve in a controlled manner over an extended depth range (∼3–20 μm using an oil immersion objective with 1.4 NA). Their complexity precludes simple Gaussian fitting; instead, maximum likelihood estimation (MLE) with a calibrated PSF model^35,36^ (Figure 2c) is required.

### 2.2 Key Challenges in SMLM Analysis before Deep Learning

Prior to deep learning approaches, SMLM reconstruction relied on detecting candidate emitters and fitting a PSF model using least-squares or maximum likelihood estimation (MLE). While effective for sparse frames, this strategy becomes prohibitively slow at high emitter densities—conditions that are otherwise desirable for reducing acquisition time. Multiemitter fitting methods^37,38^ and compressed sensing approaches^39^ extended this framework to handle overlapping PSFs. However, their computational cost scales poorly with emitter density, creating a fundamental trade-off between imaging speed and reconstruction throughput. In addition, these methods require manual tuning of parameters (e.g., density thresholds, regularization weights, PSF width) for each data set, demanding substantial user expertise and iterative adjustment.

Another fundamental challenge concerns dynamics: because SMLM reconstructions rely on aggregating thousands of sparse frames, acquisition times extend to minutes, leading to temporal averaging and motion-induced artifacts that hinder accurate capture of fast processes. More fundamentally, spatial and temporal resolutions are intrinsically coupled, imposing a spatiotemporal trade-off in which improving temporal resolution reduces localization density and degrades reconstruction quality, precluding simultaneous optimization of both.

## 3 Localization Microscopy in the Age of Deep Learning

The introduction of deep learning into localization microscopy marked a paradigm shift in super-resolution image reconstruction. Neural networks offered a new path: once trained, they can process orders of magnitude more data, far faster, and handle high emitter-densities, as no explicit emitter-fitting is occurring.

The first demonstration of this principle was Deep-STORM,^1^ which employs a fully convolutional encoder-decoder network that takes a stack of raw diffraction-limited frames and directly outputs a super-resolved image, bypassing explicit localization (Figure 3a). The network is trained on simulated data using a loss function that rewards image reconstruction accuracy, namely, the super-resolved positions of the simulated emitters, while encouraging a sparse output, and with GPU acceleration achieves reconstruction speeds of ∼ 20,000 emitters per second, which was one to three orders of magnitude faster than leading methods at the time, while delivering comparable or better reconstruction quality at emitter densities up to ∼ 6 emitters/μm^2^. Generating realistic training data is straightforward given a PSF model and estimated imaging conditions.

**Figure 3:**
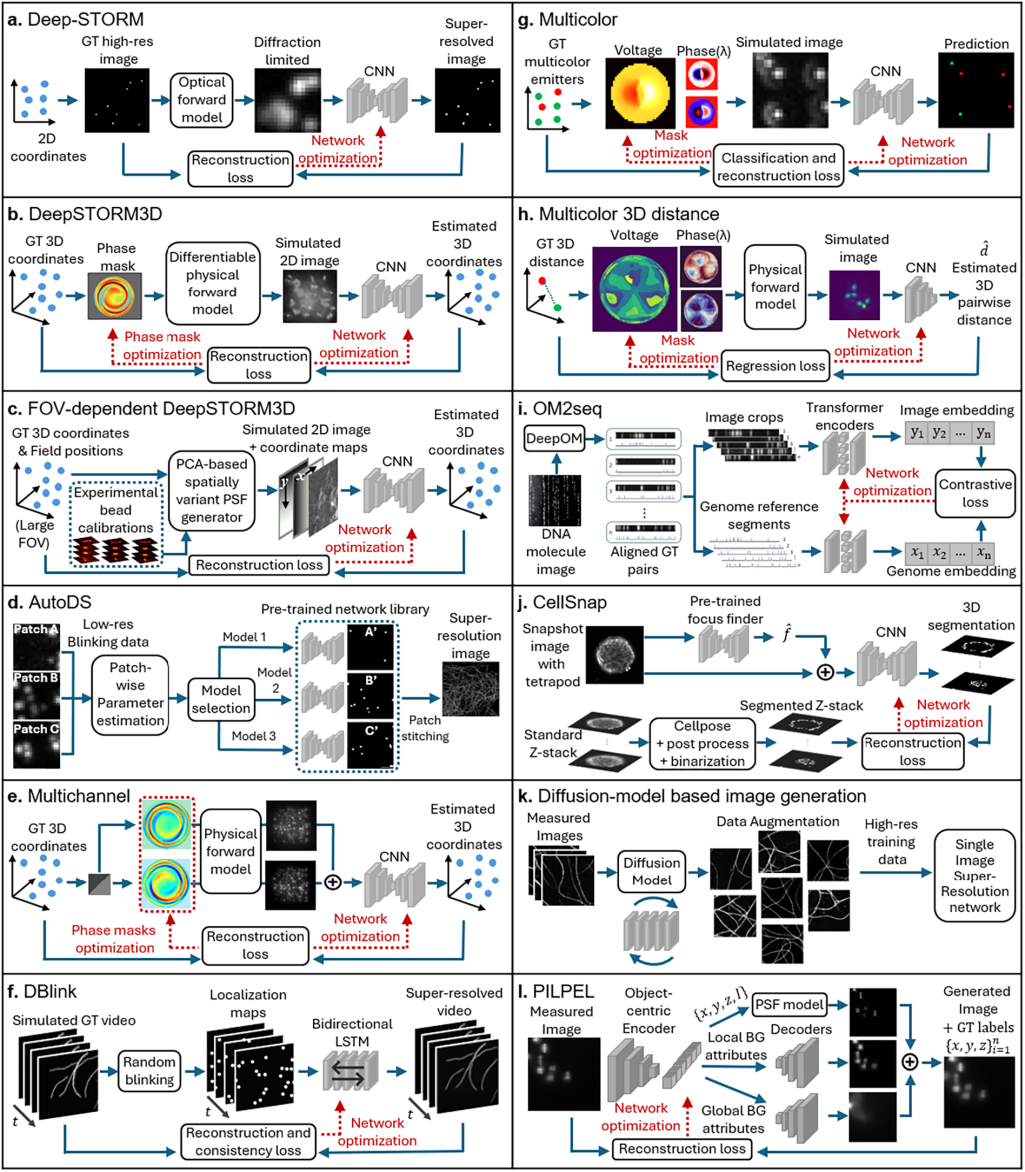
Overview of deep learning applications and training schemes in single-molecule localization microscopy. (a) Deep-STORM^1^ directly outputs a super-resolved image from raw frames, trained on simulated data using a loss that combines image fidelity and sparsity. Adapted with permission from ref 1. Copyright 2018 Optica Publishing Group. (b) DeepSTORM3D^3^ extends this to dense 3D localization. By incorporating a differentiable physical simulation layer, it also allows end-to-end joint optimization of the encoding phase mask and the decoding neural network. Adapted with permission from ref 3. Copyright 2020 Springer Nature. (c) To address optical aberrations across large fields of view, a principal-component-analysis-based spatially variant PSF generator creates simulated images concatenated with coordinate maps to train a field-aware localization network.^40^ Adapted with permission from ref 40. Copyright 2024 The American Association for the Advancement of Science. (d) AutoDS^41^ eliminates the need for postexperiment retraining in 2D SMLM by automatically extracting local imaging parameters from data patches to select the best-matching model from a pretrained library. Adapted with permission from ref 41. Copyright 2025 Springer Nature. (e) For highly dense 3D environments, a split-channel configuration uses end-to-end learning to jointly optimize two phase masks and a localization network, with each channel encoding complementary 3D information.^42^ Adapted with permission from ref 42. Copyright 2021 IEEE. (f) DBlink^4^ reconstructs superspatiotemporally resolved videos from localization data, by training a bidirectional LSTM architecture on simulated videos to capture long-range temporal dependencies in fast dynamics. Adapted with permission from ref 4. Copyright 2023 Springer Nature. (g) To achieve simultaneous multicolor localization, a wavelength-dependent phase mask is jointly optimized with the network to maximize color distinguishability and localization.^2^ Adapted with permission from ref 2. Copyright 2019 Optica Publishing Group. (h) A dual-color mask and a reconstruction network are jointly optimized to output the 3D pairwise distance between two differently colored emitters from a single shot, bypassing per-emitter localization.^43^ Adapted with permission from ref 43. CC BY 4.0. (i) OM2seq^44^ trains transformer-encoders to map DNA-fragment images and reference-genome segments into a shared embedding space, trained using a contrastive loss function, enabling fast and accurate mapping of fluorescently labeled DNA fragment to a reference genome. Adapted with permission from ref 44. CC BY 4.0. (j) A single Tetrapod-PSF widefield 2D snapshot is mapped by CellSnap^45^ directly to a 3D segmentation grid of cell nuclei, replacing z-stack acquisition. Adapted with permission from ref 45. Copyright 2024 Springer Nature. (k) A diffusion model trained on real experimental images synthesizes highly realistic data,^46^ acting as a data augmentation tool to train downstream networks, e.g., single-image super-resolution networks. Adapted with permission from ref 46. CC BY 4.0. (l) PILPEL^47^ employs a self-supervised, object-centric framework with an explicit physical PSF model in its decoder, acting as a high-fidelity data generator that intrinsically learns experimental background and noise characteristics. GT denotes Ground Truth. Adapted with permission from ref 47. Copyright 2026 The Authors.

DeepSTORM3D^3^ extended the framework to high-density 3D localization over large axial ranges using engineered PSFs (Figure 4). A convolutional-neural-network (CNN) receives a 2D frame of overlapping PSFs and outputs a full 3D localization volume, demonstrated with the 4 μm-range Tetrapod PSF. Later, other deep learning approaches to 3D SMLM were also emerging, notably DECODE,^48^ which adopts a probabilistic framework that outputs per-emitter Gaussian uncertainty estimates and has become another widely used tool in the field. A unique contribution of DeepSTORM3D was the concept of PSF codesign: by incorporating a differentiable physical simulation layer representing the phase mask, the PSF and the localization network are jointly optimized end-to-end (Figure 3b). The learned PSF (Figure 2b) outperforms the Tetrapod at high emitter densities by adopting a smaller lateral footprint that reduces interemitter overlap. Experimentally, DeepSTORM3D achieved ∼ 40 nm lateral and ∼ 50 nm axial resolution in fixed cells, and enabled volumetric telomere tracking in live cells at 10 Hz over 50 s.

**Figure 4:**
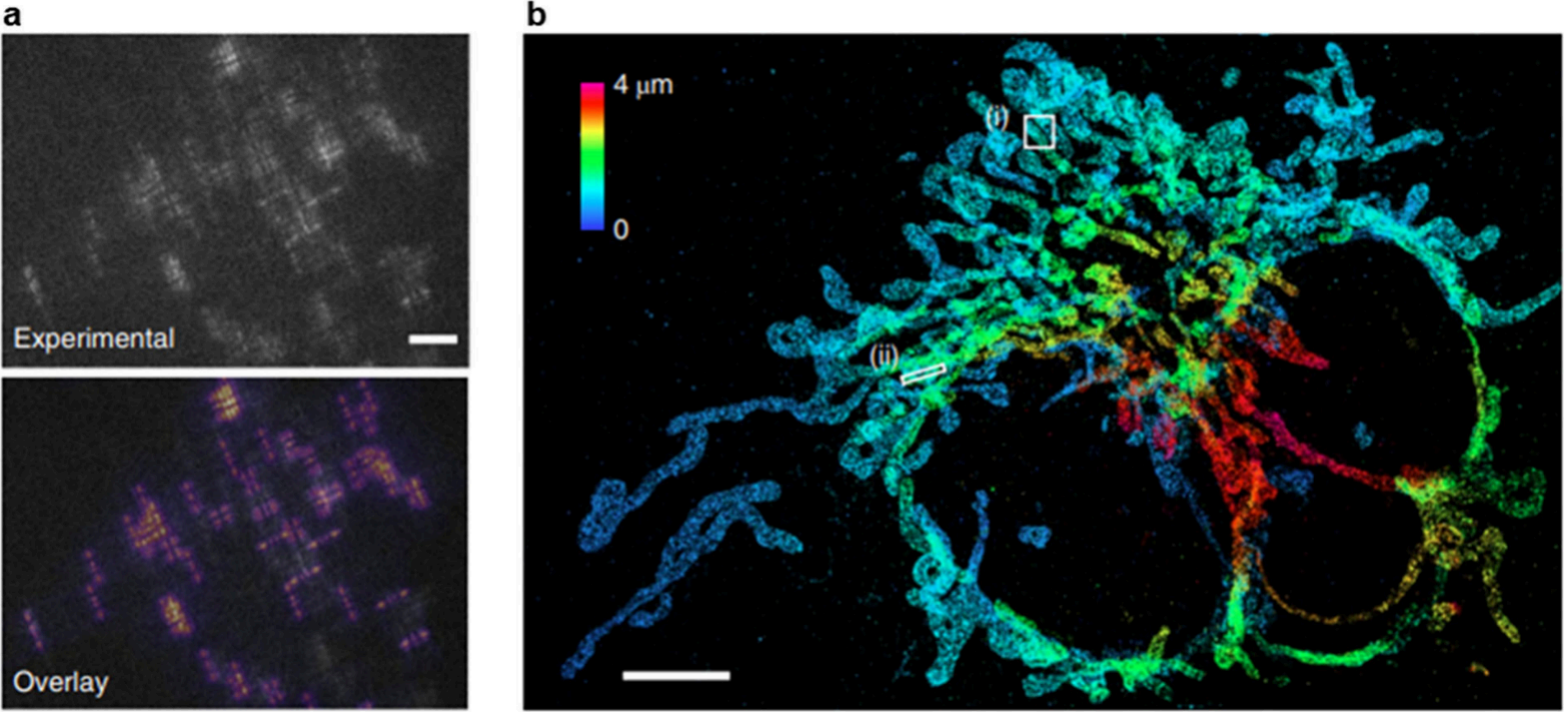
3D super-resolution imaging across a 4 μm axial range. (a) An example of raw experimental frame (top) alongside the same image overlaid with the rendering produced from the 3D emitter positions recovered by the DeepSTORM3D network (bottom). Scale bar, 5 μm. (b) Reconstructed super-resolution image of mitochondria covering 4 μm in depth, displayed as a 2D histogram with color-coded z position. Scale bar, 5 μm. Adapted with permission from ref 3. Copyright 2020 Springer Nature.

Standard localization algorithms assume a spatially invariant PSF, but this assumption breaks down at large fields of view, where optical aberrations and phase mask misalignment, particularly in compact mask insertion configurations,^45,49^ cause the PSF shape to vary with lateral position. One approach to address this is FD-DeepLoc,^50^ which combines DECODE with CoordConv layers (x and y maps of the FOV) to make the localizer aware of each emitter’s field position. A complementary approach, PPG3D,^40^ is a principal-component-analysis-based spatially variant PSF generator that interpolates calibrated PSFs across the FOV and achieves higher accuracy than existing generators while running orders of magnitude faster at the time. Combining PPG3D with a CoordConv-augmented DeepSTORM3D produces a FOV-dependent localizer trained with awareness of each emitter’s field position (Figure 3c). In simulation, this approach improves lateral and axial localization accuracy by 36% and 30% respectively over standard DeepSTORM3D, and was validated experimentally on SMLM imaging of mitochondria and microtubules over a ∼ 178 × 178 μm FOV (Figure 5).

**Figure 5:**
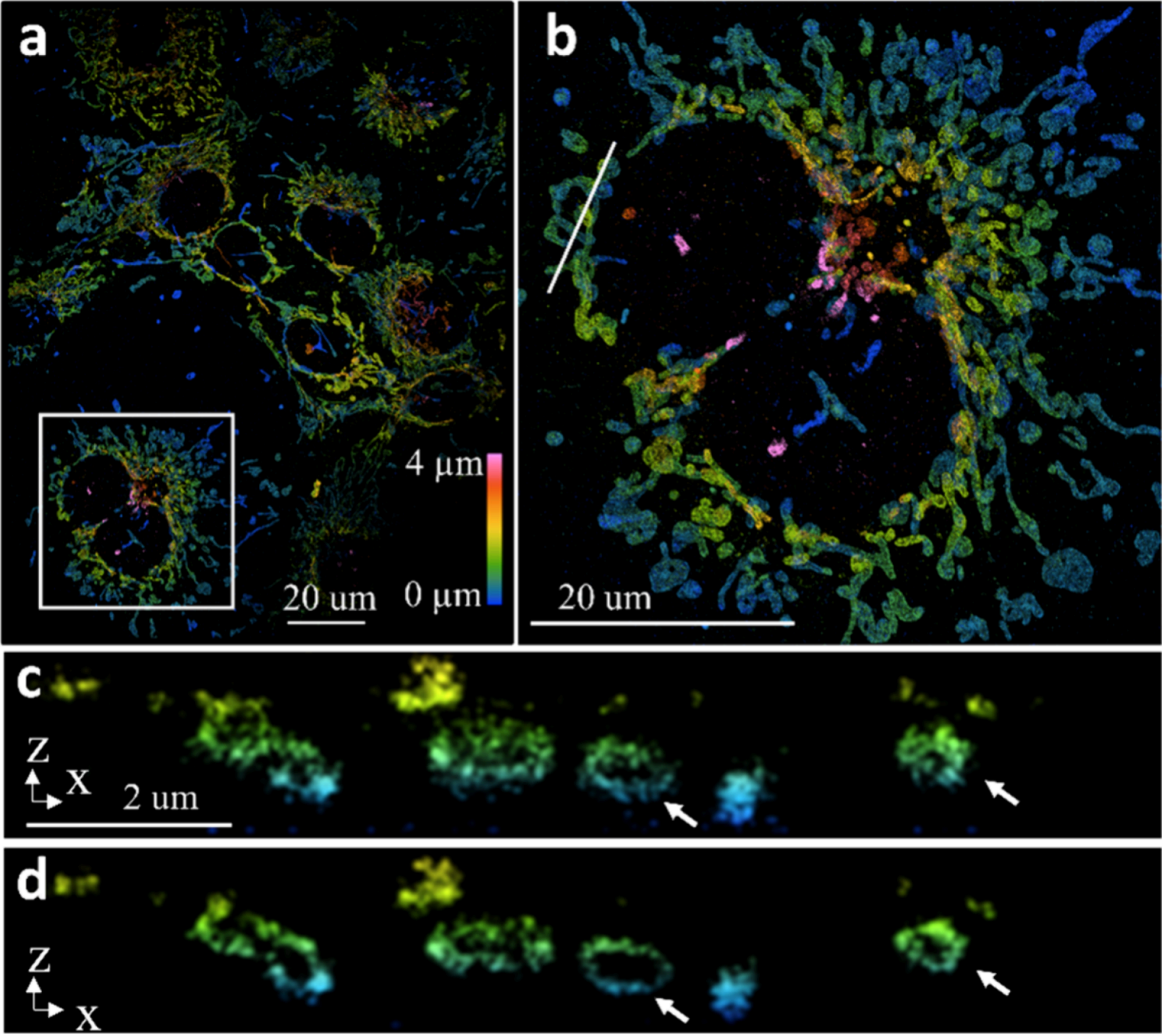
Large-FOV 3D super-resolution imaging of mitochondria with Tetrapod PSF. (a) Super-resolved 3D mitochondria image (maximum projection) in a 127-by-160 μm^2^ field of view (FOV). (b) Zoom-in view of the square region indicated in (a). (c, d) x-z cross sections along the white line in (b), comparing reconstructions from standard DeepSTORM3D (top) and FOV-dependent DeepSTORM3D (bottom). The white arrows indicate noteworthy structural details. Adapted with permission from ref 40. Copyright 2024 The American Association for the Advancement of Science.

Deep learning-based SMLM methods, being simulation-based, carry a practical bottleneck: manual tuning of simulation parameters to match each new experimental condition requires domain expertise and hours of retraining per data set. This problem is addressed with AutoDS and AutoDS3D.^41^ In 2D, AutoDS divides the FOV into patches, automatically extracts imaging parameters (emitter density, SNR, background) per patch, and selects the best-matching model from a pretrained Deep-STORM library (Figure 3d). This has the huge benefit of in-principle eliminating post-experiment training entirely. This patch-wise strategy also handles spatially heterogeneous data and outperforms a single globally trained model across all quantitative metrics. In 3D, AutoDS3D provides a one-click GUI that automates the entire DeepSTORM3D pipeline from PSF characterization to final reconstruction, completing ∼ 7 times faster than the manual workflow. Together, these tools represent a significant step toward making deep learning-based SMLM accessible to nonexpert users.

Over time, DeepSTORM3D has been extended to more complex end-to-end designs. In low-light applications like SMLM, the prevailing approach has traditionally been to condense signal photons into a single imaging channel to maximize the signal-to-noise ratio. However, for extremely dense emitters in 3D, a single-channel configuration fundamentally limits detectability due to the severe overlap of point spread functions. To overcome this, the system can be expanded into a multichannel configuration where the emission path is split into parallel channels.^42^ Each channel is modulated by its own phase mask, and these masks are jointly optimized alongside the localization neural network to maximize information extraction (Figure 3e). This split-signal system allows each channel to encode complementary 3D information to better disentangle overlapping emitters. Then, end-to-end learning can jointly optimize two phase masks alongside the localization neural network. The resulting PSFs, termed the Nebulae PSFs, encode 3D information in both channels simultaneously rather than separating lateral and axial roles. At extreme emitter densities, this end-to-end learned two-channel approach effectively doubles the detection rate and improves precision compared to the best single-channel equivalent, because reducing PSF lateral overlap between channels outweighs the per-channel photon penalty. The Nebulae PSFs enabled continuous scan-free 3D tracking of dense telomeres in live cells.^42^

Beyond 3D spatial localization, neural networks have also transformed how we extract spectral information. It is important to note that a microscope’s standard PSF inherently depends on the emission wavelength due to diffraction and chromatic aberrations. Thus, distinguishing colors from a standard PSF on a grayscale camera was already physically possible, and a neural network can exploit this subtle chromatic dependence to successfully classify these colors with no additional hardware modification.^2^ However, when simultaneously imaging more than two types of emitters, standard PSF classification becomes significantly more challenging. To address this, joint optimization of the PSF and the neural network has been used to encode spectral information directly into the PSF shape, introducing wavelength-dependent aberrations that explicitly differentiate each color (Figure 3g). By learning an optimized, wavelength-dependent phase mask, the network maximizes color distinguishability while preserving localization precision, enabling simultaneous identification and localization of up to four spectrally distinct species in a single image.^2^

Taking this a step further, more task-specific PSF engineering can be conceived. Instead of reconstructing a general-purpose super-resolved image, the entire optical and computational pipeline can be optimized end-to-end to directly output the information of biological interest. Specifically, let us consider the problem of 3D pairwise distance estimation between two differently colored molecules in a single shot,^43^ e.g. measuring the distance between two fluorescently labeled DNA loci in a live yeast cell. In this scenario, the global positions of the loci, with respect to the coverslip, are mostly meaningless; it is the distance between the loci that is of interest. By designing the PSF and the reconstruction algorithm jointly for this specific readout, intermediate localization steps are bypassed entirely (Figure 3h). This targeted approach achieves higher distance-estimation precision than traditional pipelines that first localize each emitter independently and compute distances post hoc, representing a conceptual shift from general-purpose localization toward task-specific optical computing.

The paradigm of directly mapping raw optical data to higher-order biological or structural information has also proven powerful across other imaging applications. Similar principles were applied to optical genome mapping (OGM),^51^ in which linearized DNA molecules are fluorescently labeled at specific sequence motifs, imaged, and then computationally aligned to a reference genome. In DeepOM,^52^ a CNN trained on simulated DNA images enables the resolution of closely spaced fluorescent labels that would be indistinguishable by standard nonoverlapping localization approaches, substantially improving alignment success rates for short molecules where label count is limited. This was then extended in OM2Seq,^44^ which replaces the two-step localize-then-align pipeline with a pair of Transformer-encoders, one for images and one for genome sequences, that map DNA fragment images and reference genome segments into a shared embedding space (Figure 3i). Rather than training on simulated data as in DeepOM, OM2Seq is trained on real acquired images, using a contrastive learning strategy in which very long molecules, alignable with near-100% confidence by existing methods, serve as ground truth, with shorter fragments digitally cropped from them inheriting known genome positions automatically. Reference genome segments are precomputed into a vector database, enabling fast retrieval at inference time, 2 orders of magnitude faster than prior methods.

This direct ″image-to-information″ mapping approach is highly effective for complex, volumetric biological challenges, that go beyond imaging of point-sources, such as high-throughput (HTP) wide-field microscopy of 3D tissue models like tumor spheroids. Traditionally, acquiring 3D structural data from these thick samples requires time-consuming axial scanning (z-stacks), which not only reduces throughput but also increases photobleaching and photodamage. To bypass this, a compact PSF engineering module has been implemented directly within the objective lens back focal plane to minimize the hardware footprint inside an incubator-housed HTP microscope.^45^ This modification offers versatile solutions: it can optically extend the effective depth of field for immediate z-projections without postprocessing, or, when utilizing a depth-encoding Tetrapod PSF, it pairs seamlessly with a custom deep neural network called CellSnap. CellSnap leverages this direct mapping by taking a single 2D snapshot and directly outputting a canonical 3D segmentation of cell nuclei in the form of a binary occupancy grid (Figure 3j). By eliminating the need to acquire and process multiframe 3D data cubes, this single-shot approach roughly matches the segmentation accuracy of standard z-stack acquisitions (Figure 6), but is an order of magnitude faster in both acquisition and postprocessing. In addition, this work highlights the broader principle that high-throughput imaging platforms can serve as highly efficient sources of experimental training data for deep learning.

**Figure 6:**
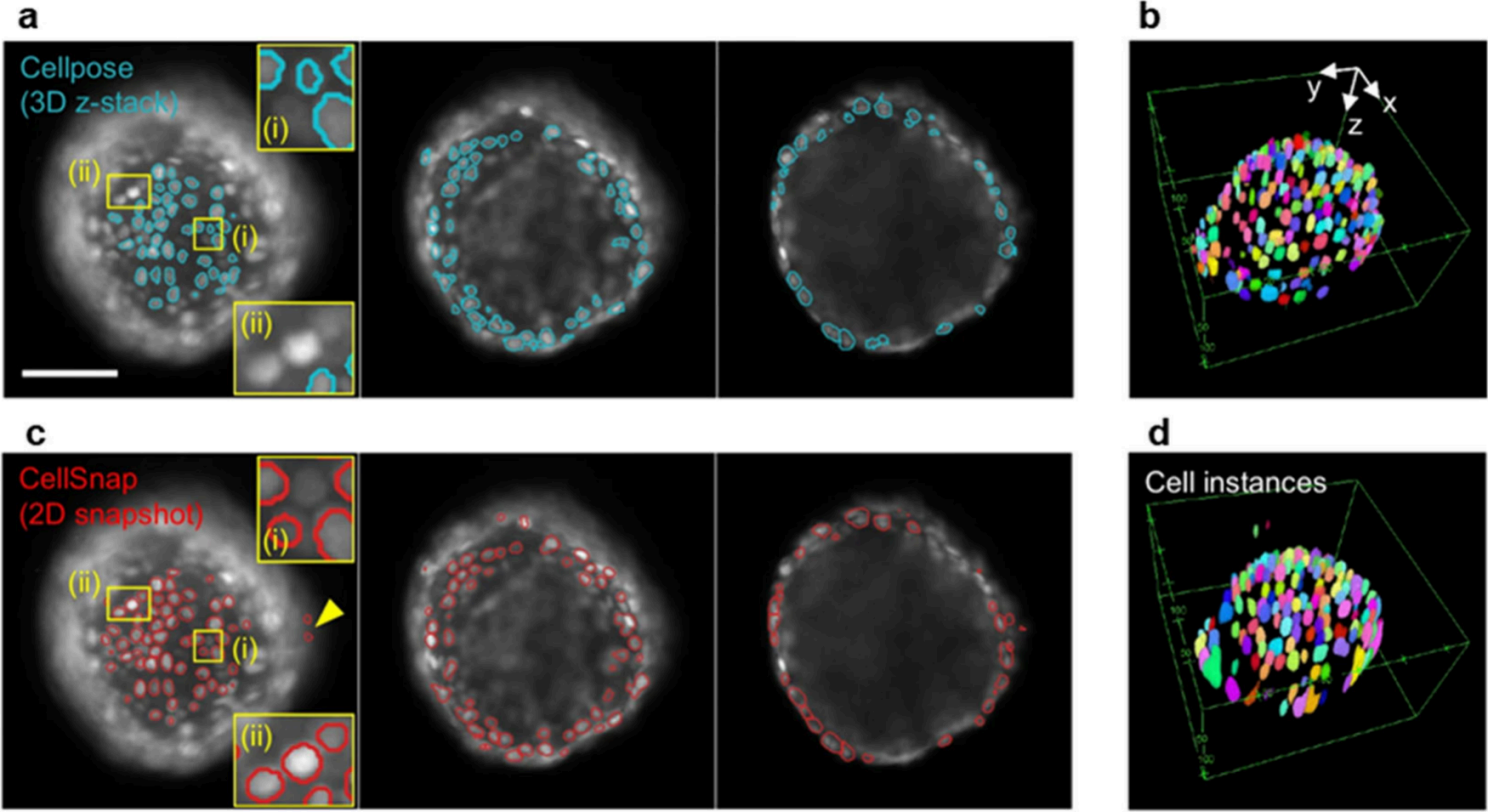
Visual comparison of CellSnap and Cellpose segmentation performance. (a) Cellpose segmentations, from a z-stack, superimposed on raw axial image slices taken at 40 μm intervals. Scale bar, 100 μm. (b) 3D rendering of discrete cell instances detected by Cellpose. (c) CellSnap segmentations, from a single snapshot, superimposed on the corresponding raw axial slices at 40 μm intervals. Yellow arrowhead indicates a detaching cell on the periphery of the axial range identified by CellSnap and missed by the conventional z-stack method. (d) 3D rendering of individual cell instances identified by CellSnap, following watershed postprocessing. Adapted with permission from ref 45. Copyright 2024 Springer Nature.

A fundamental limitation of SMLM, and in fact, of any super-resolution microscopy method, is inherently poor temporal resolution, caused by the trade-off between time and spatial resolution. Because super-resolved images are built from the accumulation of thousands of frames, each containing only a sparse subset of active emitters, a single reconstruction typically requires (at least) minutes of acquisition, precluding observation of rapid dynamics. This issue is addressed in DBlink,^4^ a deep-learning method for super spatiotemporal resolution reconstruction from SMLM data. DBlink takes as input a recorded SMLM video and outputs a super-resolved video reconstruction. It combines a convolutional neural network with a bidirectional long short-term memory (LSTM) architecture specifically designed to capture long-range temporal dependencies across frames, drawing information from both past and future frames (Figure 3f). Demonstrated on simulated and experimental data including dynein motors moving on microtubules and live-cell SMLM (Figure 7), DBlink recovers spatiotemporal information at temporal resolutions as fine as 15 ms (66.6 fps), far beyond what conventional SMLM accumulation permits, representing a significant advance in super-resolution imaging of dynamic processes.

**Figure 7:**
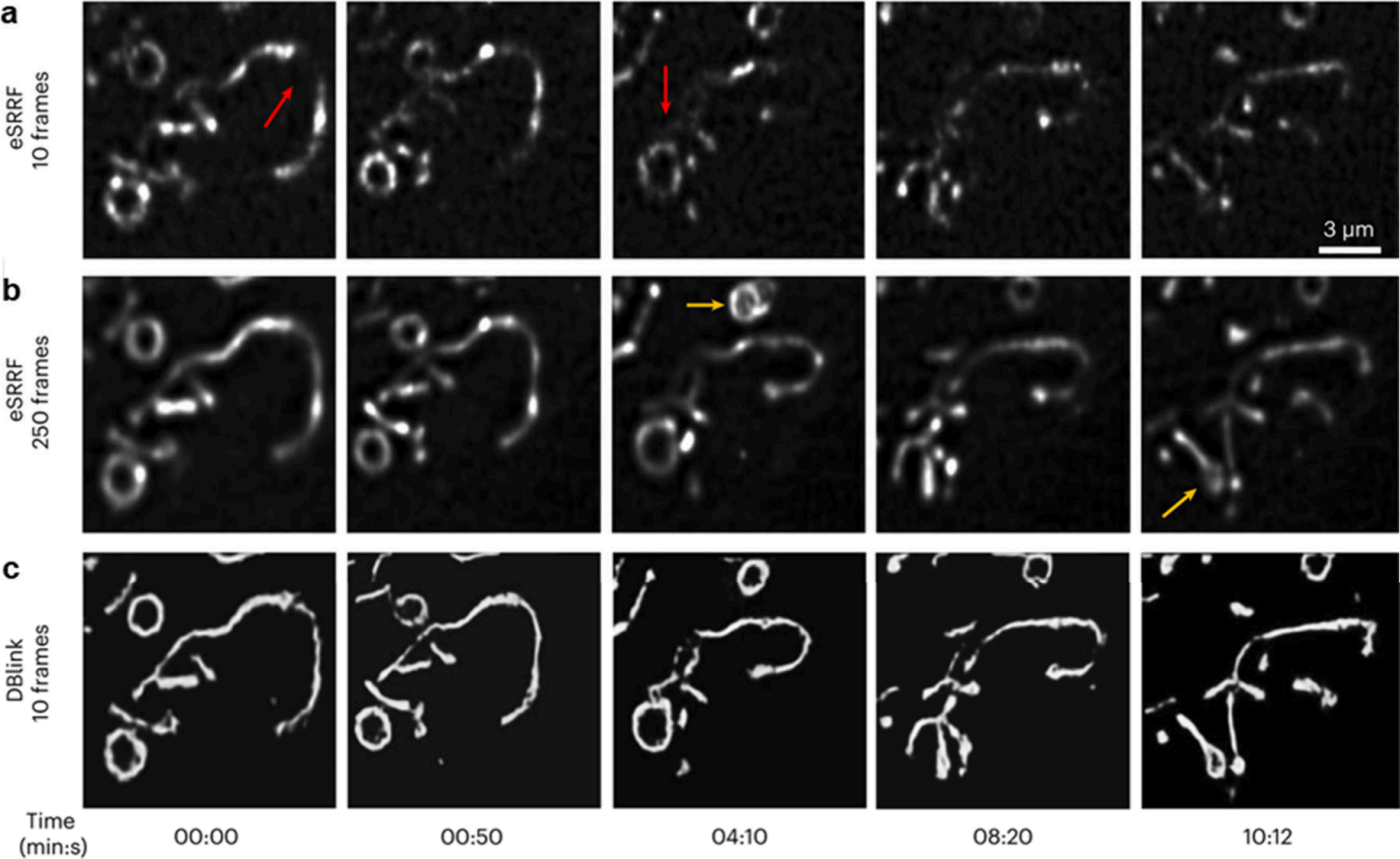
Reconstructions of live-cell mitochondrial dynamics (shown across columns) demonstrating DBlink ability to overcome the traditional spatiotemporal resolution trade-off. (a) and (b) illustrate the limitations of the alternative approach eSRRF (enhanced super-resolution radial fluctuation): utilizing long, 250-frame temporal windows introduces severe motion blur that obscures rapidly moving structures (yellow arrow), while shorter windows result in missing structural details (red arrows). (c) DBlink reconstruction showing successfully resolved continuous, high-fidelity structures at the native 20 fps temporal resolution of the blinking data without motion-blur artifacts. Adapted with permission from ref 4. Copyright 2023 Springer Nature.

A crucial aspect for the success of deep-learning methods, as mentioned earlier, is the quality of training data. Supervised networks require large data sets that accurately reflect experimental conditions, yet generating such data through simulation demands careful manual tuning of parameters and mathematical models. In “This Microtubule Does Not Exist”^46^ diffusion models, i.e. generative networks that learn to produce realistic images, trained on real experimental STED and SMLM images, were shown to synthesize highly realistic super-resolution microscopy images and serve as an effective data augmentation tool (Figure 3k). Although these generated images are inherently unlabeled, they serve as excellent training data for downstream tasks that do not require explicit ground-truth annotations, such as single-image super-resolution. The utility of this generative approach was validated by training a single-image-super-resolution (SISR) network on the synthetic data, which outperformed models trained on standard mathematical simulations across multiple quantitative metrics, illustrating the potential of generative AI for microscopy data synthesis.

To generate training data with maximal resemblance to the experimental images that the net is expected to reconstruct, PILPEL (Physics-Informed Latent Particles for Emitter Localization)^47^ merges a generative capability with explicit physical modeling. PILPEL uses a self-supervised, object-centric framework equipped with a physics-informed decoder. By incorporating an explicit physical PSF model into its decoder, PILPEL acts as a high-fidelity data generator that intrinsically captures experimentally learned background features and noise characteristics while producing precise, ground-truth emitter labels (Figure 3l). Across simulations and real biological data sets (Figure 8), training on PILPEL-generated data substantially improved localization accuracy and emitter detection rates compared to models trained on standard simulated data, with the most pronounced gains observed under challenging conditions such as complex backgrounds and low signal-to-noise ratios. These generative tools represent a shift from manual simulation engineering toward data-driven, automatically adapted training pipelines that can generalize to novel biological systems without expert intervention.

**Figure 8:**
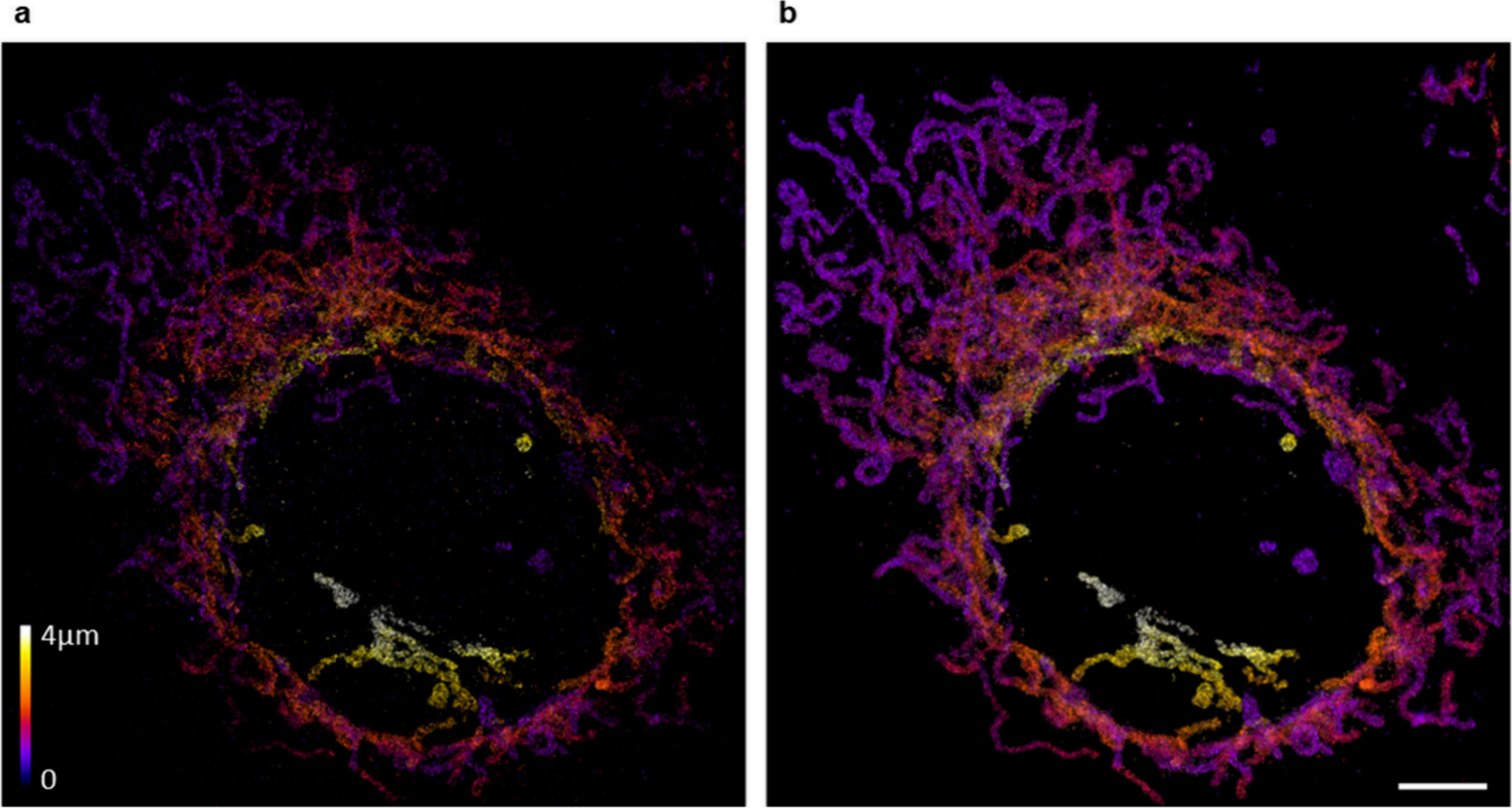
**PILPEL:** Comparison of 3D super-resolution reconstructions of experimental data. Super-resolved reconstructions of mitochondria (depth ≈ 4 μm), rendered as 2D histograms with color-coded z-positions, showing (a) the standard DeepSTORM3D reconstruction and (b) the PILPEL-trained Deep-STORM3D reconstruction. Scale bar, 5 μm. Adapted with permission from ref 47. Copyright 2026 by the authors.

## 4 Conclusion and Outlook

The integration of deep learning with SMLM represents a powerful paradigm shift in super-resolution imaging. While traditional SMLM achieved nanoscale resolution by temporally separating fluorophore emissions, conventional reconstruction algorithms are often computationally prohibitive and require careful parameter tuning, especially at high emitter densities. Deep neural networks overcome these limitations by learning directly from data, offering exceptionally fast, parameter-free inference. As discussed, this combination has unlocked new capabilities that were previously unattainable, from the end-to-end joint optimization of phase masks and localization networks to extracting dense 3D multicolor information and observing rapid spatiotemporal dynamics.

Looking forward, several notable key challenges and opportunities are currently outstanding in neural-net based localization microscopy.

### 4.1 Real-Time Processing

While neural networks significantly accelerate inference compared to traditional fitting, pushing these algorithms to operate in true real-time during image acquisition remains an ongoing goal.^53–55^ Real-time reconstruction and feedback would allow researchers to dynamically adjust imaging parameters or track fast biological processes on the fly.

### 4.2 Bridging the Simulation-to-Experiment Gap

Deep learning models heavily rely on the quality of their training data. The discrepancy between idealized mathematical simulations and real-world noisy, heterogeneous experimental data limits model performance. Future work must continue to develop and improve generative and physics-informed frameworks,^56,57^ like PILPEL, to synthesize highly realistic training sets that intrinsically capture complex background features and noise characteristics without manual tuning.

### 4.3 Development of Foundation Models

Currently, many networks require retraining or fine-tuning for specific experimental conditions, densities, or engineered PSFs. The development of training-free foundation models for microscopy,^58–61^ capable of generalizing across different microscopes, fluorophores, and optical setups out-of-the-box, would drastically increase the accessibility of these advanced techniques.

### 4.4 Reconstruction Beyond Point Sources

Finally, while SMLM inherently relies on point-source emitters, the deep-learning reconstruction principles developed here have massive potential for more complex structures. Expanding these end-to-end, direct ″image-to-information″ mapping frameworks to efficiently resolve continuous, nonpoint-source modalities and thick 3D tissue models represents a promising frontier for bioimaging.^62–65^

Ultimately, the continued convergence of novel optical engineering and artificial intelligence will improve all aspects of SMLM and super-resolution microscopy in general, from imaging performance to accessibility.
